# A Survey of Radiomics in Precision Diagnosis and Treatment of Adult Gliomas

**DOI:** 10.3390/jcm11133802

**Published:** 2022-06-30

**Authors:** Peng Du, Hongyi Chen, Kun Lv, Daoying Geng

**Affiliations:** 1 Department of Radiology, Huashan Hospital, Fudan University, Shanghai 200040, China; pdu20@fudan.edu.cn (P.D.); lvkun85093@163.com (K.L.); 2 Center for Shanghai Intelligent Imaging for Critical Brain Diseases Engineering and Technology Research, Shanghai 200433, China; 3 Academy for Engineering and Technology, Fudan University, Shanghai 200433, China; 20110860021@fudan.edu.cn

**Keywords:** radiomics, precision medicine, adult gliomas, central nervous system, individualized diagnosis and treatment, prognosis

## Abstract

Glioma is the most common primary malignant tumor of the adult central nervous system (CNS), which mostly shows invasive growth. In most cases, surgery is often difficult to completely remove, and the recurrence rate and mortality of patients are high. With the continuous development of molecular genetics and the great progress of molecular biology technology, more and more molecular biomarkers have been proved to have important guiding significance in the individualized diagnosis, treatment, and prognosis evaluation of glioma. With the updates of the World Health Organization (WHO) classification of tumors of the CNS in 2021, the diagnosis and treatment of glioma has entered the era of precision medicine in the true sense. Due to its ability to non-invasively achieve accurate identification of glioma from other intracranial tumors, and to predict the grade, genotyping, treatment response, and prognosis of glioma, which provides a scientific basis for the clinical application of individualized diagnosis and treatment model of glioma, radiomics has become a research hotspot in the field of precision medicine. This paper reviewed the research related to radiomics of adult gliomas published in recent years and summarized the research proceedings of radiomics in differential diagnosis, preoperative grading and genotyping, treatment and efficacy evaluation, and survival prediction of adult gliomas.

## 1. Introduction

Glioma is the most common primary malignant tumor of the adult CNS. Due to its invasive growth, most patients will recur even after combined treatments such as surgery, chemotherapy, and radiotherapy. With the continuous development and great progress of molecular genetics and biology technology, people have a deeper understanding of the occurrence and development of glioma, and more and more molecular biomarkers have been proved to play important roles in the classification, typing, and treatment and prognosis of glioma [1]. In 2016, the fourth revision of the WHO Classification of Tumors of the CNS introduced molecular phenotypes based on histology for the first time and proposed the concept of integrated diagnosis [2]. After five years of practice and improvement, the WHO classification of tumors of the CNS in 2021 was formulated based on integrating the latest research progress and the 7 updates of the Consortium to Inform Molecular and Practical Approaches to CNS Tumor Taxonomy-Not Official WHO (cIMPACT-NOW), focusing on promoting the application of molecular diagnosis in the classification of CNS tumors [3].

With the rapid development of radiology, some new imaging technologies have been applied to the clinic, which has improved the accuracy of preoperative grading diagnosis of glioma. However, the correct prediction of tumor molecular typing is still the bottleneck of traditional radiology. Gliomas of the same histological grade may have radically different prognoses due to different molecular phenotypes, which are difficult to identify from preoperative conventional radiology and can only rely on histopathology and molecular detection after surgical resection [4]. Nevertheless, surgery is an invasive clinical method, and the prognosis of glioma is closely related to the extent of surgical resection. If accurate histological grading and molecular phenotype of gliomas can be obtained non-invasively before surgery, it can assist with formulating individualized surgical treatment and improving the prognosis of patients maximally, which is of self-evident significance in the era of precision medicine.

**Figure 1:**
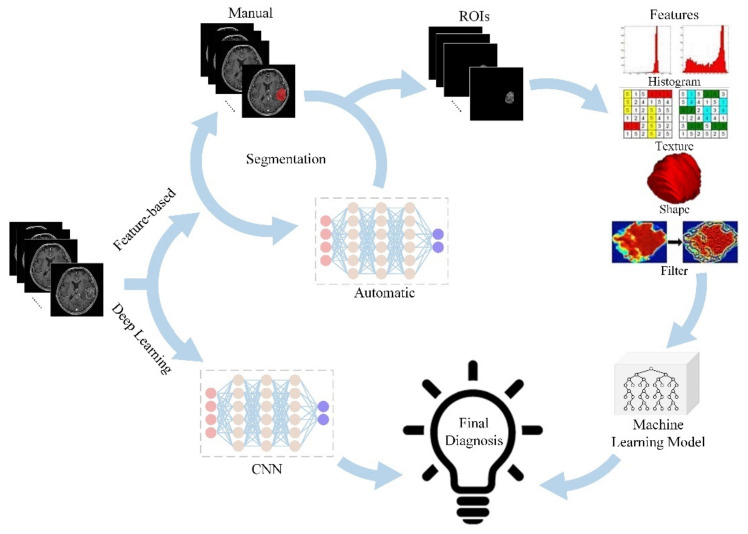
General framework showing the main steps of the radiomics.

The concept of radiomics was first proposed by Dutch scholar Lambin in 2012 [5]. It is an emerging discipline that combines traditional radiology, big data analysis, and precision medicine, making use of radiomics features extracted from massive medical images data that can be recognized and quantified by computers, which truly realizes the leap from qualitative diagnosis to quantitative analysis of traditional radiology. Radiomics is a methodology to extract a large number of image-based features from standard medical images and then apply them to clinical decision supporting systems to assist diagnosis, assess prognosis, and predict treatment response, which plays an increasingly crucial role in addressing the issue of clinical oncology research [6]. Figure 1 shows the general framework for radiomics.

## 2. Radiomics in the Differential Diagnosis of Adult Gliomas 

### 2.1. Differentiating High-Grade Gliomas (HGGs) from Solitary Brain Metastases (SBM)

There exist certain similarities between HGG and SBM in conventional radiology manifestations, such as irregular edge enhancement, tumor necrosis region, and peritumoral edema, which are difficult to distinguish sometimes and rely on the surgical or post-puncture pathological diagnosis. However, there is a huge difference in the way they are treated. HGGs are generally treated with surgery and postoperative adjuvant radiotherapy and chemotherapy, while SBM are mostly treated with radiotherapy (stereotactic radiosurgery or whole-brain radiotherapy). Therefore, accurate identification of the two is of great clinical significance, and radiomics has made some progress in this field (Table 1).

Chen et al. [7] selected 134 patients with glioblastomas (GBMs) and metastatic brain tumors and extracted radiomics features from MRI contrast-enhanced T1-weighted imaging (CE-T1WI) to build the differential diagnostic model. It was found that the model constructed by linear discriminant analysis (LDA) and logistic regression (LR) algorithm achieved the best classification results with an accuracy of 78%, sensitivity of 69%, specificity of 86%, and receiver operating characteristics (ROC) area under the curve (AUC) of 0.80. Bae et al. [9] retrospectively selected 159 patients with GBMs and 89 patients with solitary brain metastases (SBM), extracted radiomics features from T2-weighted imaging (T2WI) and three-dimensional (3D) CE-T1WI sequences, and obtained the optimal diagnostic efficiency of differentiation by using the deep neural network (DNN) algorithm. The AUC was 0.96, and the sensitivity, specificity, and accuracy were 91%, 88%, and 89%, respectively. Zhang et al. [11] extracted radiomics features from the MRI and ^18^F-FDG PET/CT images of 100 patients with solitary brain tumors (50 GBMs and 50 BM) and used a random forest (RF) classifier to make the prediction. They found that the diagnostic performance of the combined MRI and PET/CT radiomics model (AUC = 0.98) was better than either single radiomics model. Sartoretti et al. [15] extracted radiomics features from amide proton transfer-weighted imaging (APTWI) of 21 patients with gliomas and 27 patients with BM and established a prediction model based on a multi-layer perception algorithm for distinguishing gliomas from BM, achieving an AUC of 0.836. The study by Marginean et al. [16] retrospectively analyzed contrast-enhanced CT (CECT) images of 36 patients with solitary brain tumors (17 HGGs and 19 BM) and used MaZda software (version 5) for the texture analysis of peritumoral edema to discriminate HGGs from BM, and the optimal texture parameter Perc10 had a sensitivity of 81.0%, specificity of 85.7%, and AUC of 0.84. Although previous studies have achieved good diagnostic performance, there is still much room for improvement in sensitivity and specificity.

### 2.2. Differentiating GBMs from Primary Central Nervous System Lymphomas (PCNSLs)

It is of great significance for clinical decisions to non-invasively differentiate GBMs from PCNSLs before treatment. For patients with PCNSLs, high-dose methotrexate-based combination chemotherapy is the preferred treatment, and surgery is generally not required. However, patients with GBMs usually require tumor resection within the maximum safety range followed by adjuvant chemotherapy and radiotherapy. Generally, conventional MRI can distinguish between typical GBMs and PCNSLs, but in some cases, it is difficult to distinguish and almost always requires surgery or needle biopsy to determine. In recent years, many studies have non-invasively differentiated GBMs from PCNSLs by radiomics, and most of them have achieved good results.

Chen et al. [17] retrospectively collected pre-treatment MRI images of 30 patients with PCNSLs and 66 patients with GBMs. Firstly, they used a convolutional neural network (CNN) to automatically segment tumors from the CE-T1WI sequence and then used the improved scale-invariant feature transform (SIFT) method to extract 3D local voxel arrangement information from the segmented tumors. The SVM classifier was used for prediction, and the AUC, accuracy, sensitivity, and specificity of the independent validation cohort were 0.982, 90.6%, 80.0%, and 95.5%, respectively. Suh et al. [18] reviewed the pre-treatment MRI image of 54 patients with PCNSLs and 23 patients with atypical GBMs, extracted a total of 6366 radiomics features from multi-parameter MRI (CE-T1WI, T2WI, and T2-fluid attenuated inversion recovery (FLAIR) sequences) and multi-regions, and used RF algorithm to predict tumor classification. They found that the mean AUC of radiomics classifiers was 0.921, which was significantly higher than that of three radiologists (*p* < 0.001). The study by Bathla et al. [19] enrolled 34 patients with PCNSLs and 60 patients with GBMs. They extracted 9 different sequence-based feature combinations from pre-treatment MRI images and used 45 possible models for prediction, and the results showed that the model using the combination of apparent diffusion coefficient (ADC), T2-FLAIR, and CE-T1WI sequences features achieved the best performance, with the highest AUC reaching 0.977. Xia et al. [20] retrospectively collected pre-treatment MRI images (T2-FLAIR, ADC, and CE-T1WI sequences) of 240 patients (129 GBMs and 111 PCNSLs) to establish single-sequence and multi-sequences-based radiomics models, and then combined the best performing radiomics model with the diagnosis of radiologists to build a comprehensive model. It was indicated that the radiomics model on the basis of the combination of CE-T1WI and ADC has the best performance in the validation set, with an AUC of 0.943, and the comprehensive model had better diagnostic performance compared to radiologists.

## 3. Radiomics in Preoperative Grading of Adult Gliomas

The latest edition of the WHO classification of tumors of the CNS in 2021 divided gliomas into grades 1–4 from low to high grade. Low-grade gliomas include grades 1 and 2, while high-grade gliomas include grades 3 and 4. Gliomas of different grades have different characteristics of invasiveness and infiltration, and the treatment plan and prognosis vary widely [3]. Therefore, it is of great significance for the formulation of the surgical plan and the implementation of the follow-up treatment plan if an accurate prediction of gliomas grade can be obtained non-invasively before surgery. Previous preoperative grading mainly relied on the subjective judgment of radiologists and neurosurgeons based on tumor location, shape, signal, and enhancement characteristics, which lacked objectivity, resulting in inaccurate grading. In addition, gliomas often contain cells of different grades, and grading is mainly based on the tumor cells of the highest grade. However, some cells are difficult to be manually identified in conventional radiology, so it is essential to find an objective, stable, and reliable classification method. At present, a large number of radiomics studies have been involved in the preoperative grade prediction of gliomas (Table 2).

Chen et al. [21] retrospectively analyzed MRI images of 220 patients with HGGs and 54 patients with low-grade gliomas (LGGs), segmented tumor regions using a multi-scale 3D CNN, and extracted a wide range of radiomics features. The prediction model of gliomas grade constructed by SVM achieved an accuracy of 91.27%. The study by Tian et al. [22] collected preoperative MRI images of 153 patients with gliomas (42 LGGs and 111 HGGs). They extracted radiomics features from multiple sequences (T1WI, T2WI, ADC, CE-T1WI, and 3D-arterial spin labeling (ASL)) and used an SVM classifier for grading prediction. It was found that the AUC and accuracy of the multimodal MRI-based model were 0.987 and 96.8%, respectively. Jeong et al. [23] collected the dynamic susceptibility contrast magnetic resonance imaging (DSC-MRI) images of 25 patients with histopathologically confirmed gliomas (13 HGGs and 12 LGGs) and performed grade prediction using an RF classifier based on delta radiomics features, and the final AUC was 0.94. Zhang et al. [27] retrospectively analyzed MRI diffusion tensor imaging (DTI) of 108 patients with gliomas (43 LGGs and 65 HGGs) and extracted a group of new radiomics features from the fractional anisotropy (FA) and mean diffusivity (MD) maps. SVM model was assigned two prediction tasks: LGGs vs. HGGs and grade III vs. IV, and the results showed that when using FA and MD integrated radiomics features, the AUC of classifying LGGs and HGGs was 0.93, while the AUC of classifying grade III and IV was 0.99. Many studies have shown that radiomics is a promising non-invasive method for grading gliomas, and the addition of multiple modalities and sequences will likely further improve the accuracy of predictive models. However, this kind of addition may also raise the issues of standardization and consistency. Therefore, future research needs to find a standardized, simple, and reproducible imaging modality or sequence combination for preoperative grade prediction of gliomas.

## 4. Radiomics in Predicting Genotyping of Adult Gliomas

Studies have shown that there are more than 60 kinds of genetic modification of gliomas [34]. The following changes in molecular typing have been proved to be of great significance for the formulation of clinical treatment and prognosis: (1). isocitrate dehydrogenase (IDH) mutation; (2). O6-methylguanine-DNA methyltransferase (MGMT) promoter methylation; (3). 1p/19q co-deletion; (4). telomerase reverse transcriptase (TERT) promoter mutation [35]. The latest version of the WHO classification of tumors of the CNS introduced molecular diagnostic indicators, emphasizing the role of molecular typing in the classification and grading of gliomas. It is recommended that CNS tumors should undergo hierarchical diagnosis, including histopathological classification and molecular typing, which can more accurately guide the clinical plan of surgery, adjuvant radiotherapy, and chemotherapy [36]. Therefore, it is of great significance for the treatment of patients with gliomas if accurate molecular classification can be obtained before surgery. 

### 4.1. IDH Mutation 

IDH is a key rate-limiting enzyme in the tricarboxylic acid cycle, which catalyzes the oxidative decarboxylation of isocitrate to generate α-ketoglutarate and CO_2_, providing energy for cellular metabolism and precursors for biosynthesis. IDH has three isomerase forms (IDH1/2/3), of which IDH1 and IDH2 are important indicators of glioma molecular classification, which are of great significance for the diagnosis, individualized treatment, and prognosis of gliomas [37]. Studies have shown that IDH wild-type gliomas are more prone to recur than IDH-mutated gliomas [38]. The latest WHO classification of tumors of the CNS clearly states that GBMs will contain only IDH wild-type tumors and that all IDH-mutated diffuse astrocytic tumors are considered as a single type (astrocytoma, IDH-mutated) [3]. Therefore, preoperative non-invasive identification of IDH mutation status is beneficial to provide personalized and precise treatments for patients with gliomas. Previous studies have shown that radiomics models have shown great potential in predicting IDH mutation status (Table 3). 

The study by Li et al. [40] collected preoperative multimodal MRI images of 225 patients with GBMs from multi-centers, and extracted 1614 radiomics features from enhanced, non-enhanced, necrotic, edema, tumor-core, and overall-tumor regions. Models combining regional radiomics features with clinical factors (age, gender, and Karnofsky performance status) were established separately to predict IDH1 mutation status, and it was found that the model based on overall-tumor region radiomics features and age achieved the best performance with an accuracy of 97% and AUC of 0.96. Li et al. [41] retrospectively analyzed ^18^F-FDG PET/CT images of 127 patients with gliomas, extracted a series of quantitative features reflecting the heterogeneity of tumor metabolism, and constructed a combined model including clinical and radiomics features to predict IDH mutation status. The results showed that the model combining radiomics features, age, and tumor metabolism type achieved excellent performance, with AUCs of 0.911 and 0.900 in the training and validation cohorts, respectively. Park et al. [45] enrolled 168 patients with LGGs, extracted 253 and 158 radiomics features from preoperative conventional MRI (CE-T1WI, T2WI, and T2-FLAIR) and DTI, respectively, and used RF classifier to predict IDH mutation status. They found that the model with the addition of DTI radiomics features significantly improved the accuracy of IDH genotyping (AUC = 0.900) compared to the model using traditional MRI radiomics features only (AUC = 0.83). Choi et al. [50] reviewed preoperative MRI images (CE-T1WI, T2WI, and T2-FLAIR) of 1166 patients with gliomas (grade II-IV) and established a fully automated model that comprised a CNN for tumor segmentation (Model 1) and CNN-based classifier for IDH status prediction (Model 2) that used a hybrid approach based on 2D tumor images and radiomics features from 3D tumor shape and loci guided by Model 1. The accuracies of the model on the two external validation datasets were 87.9% and 78.8%, with AUCs of 0.94 and 0.86, respectively. Therefore, it is feasible to use radiomics to predict IDH mutation status. Moreover, the addition of clinical risk factors and the use of multiple imaging modalities and novel imaging sequences can improve the predictive performance of the model to a certain extent. 

### 4.2. MGMT Promoter Methylation

MGMT is a DNA repair enzyme and is mainly distributed in the cytoplasm, which repairs DNA to maintain the stability of the genome in cells. In normal tissues, the CpG site in the MGMT promoter region is generally in an un-methylated state, but with the occurrence of the tumor, its promoter region is methylated. If the MGMT promoter is methylated, it will cause the loss of MGMT expression, resulting in a decrease in DNA repair and making gliomas more sensitive to chemotherapy drugs such as temozolomide (TMZ). Therefore, MGMT promoter methylation status is an independent predictor of prognosis in patients with gliomas [51,52]. Many researchers have also paid attention to this, trying to use radiomics to non-invasively predict the methylation status of MGMT promoters (Table 4). 

The study by Xi et al. [53] reviewed preoperative routine MRI images (T1WI, CE-T1WI, and T2WI) of 98 patients with GBMs and used the SVM algorithm to build a model to predict MGMT promoter methylation status. It was found that the best model was derived from the combination of 36 radiomics features of T1WI, CE-T1WI, and T2WI sequences, with an accuracy of 80.0% in an independent validation cohort. Jiang et al. [55] extracted a total of 1702 radiomics features from preoperative 3D-CE-T1WI and T2WI images of 122 patients with LGGs and used the least absolute shrinkage and selection operator (LASSO) algorithm for feature selection. Multiple classifiers were used to build MGMT promoter methylation prediction models. They found that the model incorporating 3D-CE-T1WI and T2WI radiomics features exhibited the best performance, with AUCs of 0.970 and 0.898 in the training and validation datasets, respectively. Kong et al. [57] retrospectively enrolled 107 patients with pathologically confirmed primary diffuse gliomas and extracted a total of 1561 radiomics features from the 3D region of interest (ROI) on the standard uptake value (SUV) map generated from the original ^18^F-FDG-PET/CT. The predictive performance of models based on radiomics features, clinical features, and fusion features were compared, and it turned out that the radiomics features-based model showed the best performance, with an AUC of 0.86 in the validation cohort, outperforming the clinical and fusion features-based models. Crisi et al. [58] retrospectively analyzed the DSC-MRI of 59 patients with GBMs and obtained a total of 92 quantitative radiomics features from relative cerebral blood volume (rCBV) and relative cerebral blood flow (rCBF) maps, and then they established a model to predict the MGMT promoter methylation status, using a multilayer perceptron algorithm. The results showed that the sensitivity of the model was 75%, the specificity was 85%, and the AUC was 0.84. The studies above indicated that radiomics is a convenient and effective method to predict the methylation status of the MGMT promoter. The application of multiple imaging modalities and multimodal-based radiomics models can successfully predict the methylation status of the MGMT promoter. However, the addition of other features, such as clinical features, did not significantly improve the predictive power.

### 4.3. 1p/19q Co-Deletion

1p/19q co-deletion refers to the combined deletion of the short arm of chromosome 1 and the long arm of chromosome 19, which can occur in various glioma subtypes, with oligodendroglioma being the most common, and GBM having a very low incidence. Many studies have demonstrated that 1p/19q co-deletion status enhances tumor sensitivity to different types of treatments [61]. Therefore, gliomas with 1p/19q co-deletion are sensitive to chemotherapy and have a significantly improved prognosis. Several studies have used MRI-based radiomics features to predict 1p/19q co-deletion status in LGGs, showing good predictive performance. 

Shofty et al. [62] retrospectively analyzed preoperative MRI images of 47 patients with LGGs and extracted a total of 152 radiomics features from T2-FLAIR, T2WI, and CE-T1WI sequences, and used 17 machine learning classifiers for 1p/19q co-deletion status prediction. It was found that the Ensemble Bagged Trees classifier obtained the best classification result, with an accuracy of 87%, and an AUC of 0.87. Han et al. [63] reviewed 277 patients with histopathologically diagnosed LGGs, extracted 647 radiomics features from preoperative MRI images, and applied an RF algorithm to generate a radiomics model for 1p/19q co-deletion status prediction. Meanwhile, a clinical model composed of relevant clinical factors and an integrated model combining radiomics features and relevant clinical factors were constructed. They found that the radiomics model showed excellent performance in the training and validation cohorts, with AUCs of 0.887 and 0.760, respectively, which were better than the clinical model. Moreover, there was no significant difference in the predictive performance between the radiomics model and the combined model, indicating that the addition of clinical factors did not bring additional predictive improvement. Kong et al. [64] reviewed 3D-CE-T1WI and T2WI images of 96 patients with LGGs and generated simulated routine CE-T1WI. Three models for predicting 1p/19q co-deletion status were constructed on the basis of 107 radiomics features extracted from each imaging modality, and it was found that the 3D-CE-T1WI model had the best performance, with accuracy and AUC of 0.897 and 0.889 in the validation dataset. Park et al. [65] retrospectively analyzed the preoperative MRI images of 93 patients with WHO grade II gliomas and extracted whole tumor histograms and texture features from ADC and DTI images. It was indicated that the skewness and cluster shade of ADC, energy, and correlation of the FA were independent predictors of 1p/19q co-deletion in IDH1-mutant LGGs. Therefore, machine learning methods based on MRI radiomics can non-invasively predict the 1p/19q co-deletion status of LGGs and provide certain help for clinical decisions. Furthermore, the combined model incorporating relevant clinical factors failed to provide additional improvement in the predictive outcome.

### 4.4. TERT Promoter Mutation

TERT is a reverse transcriptase catalyzed subunit of telomerase that maintains telomere length, which is associated with unrestricted proliferation of tumor cells. TERT promoter mutation is one of the common genetic mutations in adult diffuse gliomas, which usually occurs in the promoter region -124 and -146 base pairs (C228T and C250T), which can enhance TERT transcription. TERT promoter mutation is closely associated with 1p/19q co-deletion in LGGs [66] and is also present in GBMs and some low-grade IDH wild-type diffuse gliomas. According to the latest WHO classification of tumors of the CNS, IDH wild-type astrocytoma would be diagnosed as GBM if it meets any of the criteria or combinations of TERT promoter mutation, epidermal growth factor receptor (EGFR) amplification, chromosome 7 amplification/chromosome 10 deletion, even if the histological grade is low [3]. Therefore, if accurate TERT promoter mutation status can be obtained non-invasively before treatment, it can guide doctors to make more accurate and effective treatment plans and improve the prognosis of patients to the maximum extent. Researchers have established radiomics-based classification models to predict TERT promoter mutation status in patients with gliomas, and some progress has been made.

Fang et al. [67] extracted a total of 1293 radiomics features from preoperative MRI images (T1WI, CE-T1WI, and T2WI sequences) of 164 patients with WHO grade II gliomas and built a model for predicting TERT promoter mutation status based on the 12 most valuable radiomics features selected by nested 10-fold cross-validation cycle. The results showed that the overall accuracy was 79.88%, and the AUC was 0.8446. The study by Jiang et al. [68] collected 116 patients with pathologically confirmed LGGs, and three types of ROI (tumor region, peritumoral region, tumor, and peritumoral region) were delineated on the 3D-CE-T1WI and T2WI sequences. Models based on three regional radiomics features for predicting TERT promoter mutation status were built by multiple classifiers. It was found that the tumor region-based model showed the best performance with AUCs of 0.948 and 0.827 in the training and validation cohorts, respectively. Moreover, the addition of features of the peritumoral region did not significantly improve the predictive power. Tian et al. [69] retrospectively enrolled 126 patients with HGGs and extracted radiomics features from tumor-enhanced, necrotic, and edema regions of conventional MRI and MRS. The optimal radiomics features (Radscore) were obtained by LASSO regression and the LR algorithm was used to establish models for predicting TERT promoter mutation status. It was indicated that the model including age, Cho/Cr, Lac, and Radscore achieved the best predictive performance (AUC = 0.917). Therefore, MRI-based radiomics features are reliable for the non-invasive assessment of TERT promoter mutation status in gliomas, and future studies need to explore whether the addition of more imaging modalities and sequences can further improve the predictive performance.

### 4.5. Combined Prediction of Multiple Genotypes

1p/19q co-deletion, TERT promoter mutation, ATRX mutation, and TP53 mutation combined with IDH genotype can provide a more comprehensive characterization of intra-glioma heterogeneity. Therefore, a large number of studies have constructed radiomics-based models for the combined prediction of multiple genotypes.

The combination of IDH genotype, 1p/19q co-deletion, or TERT promoter mutation status is often used to evaluate the prognosis of patients with gliomas. Lu et al. [70] proposed a multimodal MRI radiomics-based three-level machine learning model to classify five glioma molecular subtypes (based on IDH genotype and 1p/19q co-deletion status), achieving an accuracy of 81.8%. Arita et al. [71] established a radiomics model based on conventional MRI images and lesion location information to classify three molecular subtypes of grade II/III gliomas (based on IDH genotype and TERT promoter mutation status), and the accuracy of the training set was 74%. ATRX mutation and TP53 mutation are typical molecular variants of adult IDH-mutant astrocytomas and are also important auxiliary diagnostic markers [72,73]. The study by Wu et al. [74] indicated that nomogram incorporating age, gender, and radiomics features provided an effective method for non-invasively predicting IDH genotype and ATRX mutation status in patients with LGGs. Sohn et al. [75] established a radiomics-based multi-gene prediction chain model to predict IDH and ATRX mutation status of WHO grade 4 astrocytomas and obtained the highest AUCs of 0.967 and 0.822, respectively. Zhang et al. [76] used a radiomics model to classify three molecular subtypes of LGGs (based on IDH and TP53 mutation status) and achieved satisfactory results, with accuracy > 70%. In addition, the latest WHO classification of tumors of the CNS proposed that if some IDH wild-type LGGs meet the molecular characteristics of GBM, they should also be diagnosed as GBM [3]. Park et al. [77] retrospectively collected 121 patients with IDH wild-type LGGs and constructed a machine learning classifier based on preoperative MRI radiomics features to predict LGGs with molecular features of GBM, and the result was satisfied with an AUC of 0.854. Therefore, the radiomics-based models can achieve the combined prediction of key genotypes of gliomas, which can further improve the classification and integrated diagnosis of tumors, assisting doctors to select more reasonable treatment plans.

## 5. Radiomics in the Treatment and Efficacy Evaluation of Adult Gliomas

Treatment options for gliomas depend on grade, genotype, size, and location of tumors. Currently, many treatments have been tried for gliomas, including surgery, radiotherapy, chemotherapy, targeted therapy, and electric field therapy, but each treatment has its limitations. Typically, the standard treatment plan for gliomas is based on maximum safe surgical resection followed by adjuvant chemotherapy and radiotherapy, but even with strict implementation of standard therapy, a large proportion of patients relapse after treatment. Therefore, selecting a more reasonable and safe surgical resection range and early evaluation of treatment response will be beneficial to the formulation and adjustment of treatment plans, to maximize the improvement of patients’ prognosis.

### 5.1. Identification of Tumor Infiltration Region

Most adult gliomas grow invasively, and the border of the tumor cannot be accurately determined by the naked eye and conventional radiology, which makes it difficult for surgeons to achieve total resection. Therefore, some tumor tissues are often left on the resection margin, and the amount of residual tumor tissues is closely related to the prognosis of patients with gliomas [78]. Hence, it is necessary to find a non-invasive method to identify the tumor infiltration region and estimate the residual tumor region early and accurately and help formulate individualized surgical treatment plans, to guide doctors to safely remove tumors and improve the scope of resection, achieving “super total resection” and improving the outcomes of patients.

Akbari et al. [79] used a machine learning approach based on radiomics features extracted from preoperative multi-parametric MRI (T1WI, CE-T1WI, T2WI, T2-FLAIR, DTI, and DSC-MRI sequences) of 31 patients with GBMs, creating a spatial map of peritumoral tissue infiltration to predict early recurrence. In a validation set of 34 patients, the model obtained an AUC of 0.84 and a sensitivity of 91%. It was indicated that radiomics can assess the extent of GBM infiltration and predict the location of future tumor recurrence. The study by Yan et al. [80] retrospectively analyzed preoperative MRI images (structural MRI, perfusion MRI, and DTI) of 57 patients with GBMs, and extracted voxel-based radiomics features to train the CNN model for identifying the progressive region around the tumor. The model achieved overall accuracies of 92.6% and 78.5% in the training and validation sets, respectively. Meanwhile, it was found that compared with the non-progressive region, the signal intensities of FLAIR, rCBV, and CE-T1WI in the progressive region around the tumor were higher. Rathore et al. [81] established a machine learning model based on preoperative MRI radiomics to predict the recurrence area of GBM and tested 90 patients with GBMs in two cohorts. The results showed that accuracies in the two cohorts were 87.51% and 89.54%, respectively. They also found that the tumor recurrence area was characterized by dense cellular structure and vascular distribution, with hypointensity on T2WI and T2-FLAIR images. The studies above suggest that multi-parametric MRI radiomics features can help assess the degree of glioma infiltration and predict the location of future tumor recurrence, which may guide doctors to conduct precise surgery and postoperative radiotherapy, so as to delay the tumor recurrence and improve patients’ survival. However, the real application of this technology to the clinic requires to be verified by prospective, multi-centered, and large-sample studies. Meanwhile, patients with LGGs need to be added to the sample.

### 5.2. Discrimination of True Tumor Progression (TTP) and Pseudoprogression (PsP) after Chemotherapy and Radiotherapy

PsP is a relatively common subacute response to radiotherapy and chemotherapy in HGGs, occurring in approximately 20% of all patients with GBMs receiving standard treatment [82]. In both conventional radiology and clinical manifestations, PsP is strikingly similar to TTP occurring at the tumor site or the resection margin. PsP does not require treatment and resolves or remains stable over time [83,84], while TTP represents treatment failure and requires timely change or adjustment to the treatment strategy, both of which rely on surgical resection for confirmation. Therefore, if we can identify TTP and PsP non-invasively, timely, and accurately, it will be beneficial to adjust the treatment plan as soon as possible, and reduce the damage and burden caused by unnecessary treatment to patients. 

Qian et al. [85] utilized a spatio-temporal discriminative dictionary learning model based on longitudinal DTI radiomics features to distinguish between TTP and PsP in GBMs, achieving an average accuracy of 0.867 and AUC of 0.92 in the experimental group. Zhang et al. [86] used a radiomics model based on combined features and multimodal MRI images (T1WI, CE-T1WI, T2WI, and T2-FLAIR sequences) to classify radiation necrosis and recurrence in patients with gliomas, and the best performing model obtained an AUC of 0.9982. Kim et al. [87] incorporated diffusion-weighted imaging (DWI) and perfusion-weighted imaging (PWI) into a multi-parameter radiomics model to differentiate between TTP and PsP of GBMs, and the AUC of this model was higher than any single-parameter model based on conventional MRI. Patel et al. [88] used a machine learning model combining age, MGMT promoter methylation status, and MRI radiomics features, achieving the identification of early TTP and PsP in patients with GBMs (AUC = 0.80, accuracy = 73.7%). Therefore, functional MRI-based radiomics is superior to conventional MRI in distinguishing TTP from PsP of gliomas after chemotherapy and radiotherapy due to that it can provide some quantitative indicators to quantify abnormal signals around the residual cavity after surgery.

### 5.3. Judgment of Sensitivity to Chemotherapy and Targeted Drugs

TMZ is currently the first-line chemotherapy drug for gliomas, with the advantages of low toxicity and strong anti-tumor activity. Its mechanism of action is to effectively block the cell cycle of glioma cells, inhibit cell proliferation, and lead to apoptosis. TMZ has a strong ability to penetrate the blood-brain barrier, which can effectively delay the development of tumors and improve the quality of life and survival of patients [89]. Bevacizumab is a humanized monoclonal antibody that inhibits tumors by acting on vascular endothelial growth factors. It is currently a widely used targeted drug for patients with recurrent GBMs [90]. Therefore, accurate prediction of the sensitivity of the patients with gliomas to certain drugs has important reference value for formulating individualized treatment plans.

Wei et al. [56] constructed a radiomics model based on MRI images (CE-T1WI, T2-FLAIR, and ADC) and successfully classified patients with WHO grade 2–4 astrocytomas into high-risk and low-risk groups for overall survival after TMZ chemotherapy, achieving survival stratification for TMZ chemotherapy. Wang et al. [91] identified patients who were more likely to benefit from chemotherapy using radiomics features extracted from preoperative MRI (CE-T1WI and T2-FLAIR) of patients with LGGs. Kim et al. [92] analyzed MRI perfusion images of 72 patients with recurrent GBMs after standard therapy and found that patients with high blood flow in the tumor region with MGMT promoter methylation could benefit from TMZ chemotherapy. They believed that high blood flow enabled tumors to obtain higher drug concentrations, which in turn inhibited tumor growth more effectively. Kickingereder et al. [93] extracted relative blood flow volume from MRI perfusion images of patients with GBMs, and it was found that this feature was effective in predicting the sensitivity of recurrent GBMs to bevacizumab treatment, but not to TMZ chemotherapy. After that, Kickingereder et al. [94] extracted 4842 quantitative radiomics features from MRI images (T1WI, CE-T1WI, and T2-FLAIR) of 172 patients with recurrent GBMs and constructed a model that can efficiently predict the sensitivity of patients with recurrent GBMs to bevacizumab treatment using principal component analysis machine learning method. Therefore, whether radiomics features are based on single-modality or multi-modality, conventional MRI sequences or functional MRI sequences, can accurately determine the sensitivity of patients with gliomas to certain chemotherapy and targeted drugs, assisting doctors to implement individualized and precise treatment for patients.

### 5.4. Survival Prediction

With the use of novel treatments such as electric field therapy, targeted therapy, and immunotherapy, the treatment of gliomas has made some progress, but the survival rate of patients with HGGs is still very low. The median survival of patients with GBMs is approximately 12–15 months, and the five-year survival rate is less than 10%. Therefore, the use of radiomics-based methods to predict the survival of patients with gliomas has become a clinical concern. The study of Li et al. [95] indicated that the model based on radiomics features extracted from preoperative T2WI images of patients with gliomas can stably predict the survival of the patients, and radiomics features in predictive models were found to correlate with immune responses and facilitate the preoperative assessment of the extent of gliomas macrophage infiltration. Han et al. [96] combined deep features generated by pre-trained CNN with conventional radiomics features to build a model to predict overall survival in patients with HGGs. The result showed that in all three test cohorts, patients were effectively divided into long-term and short-term survivors. Yan et al. [97] used radiomics models based on preoperative MRI (CE-T1WI and ADC), achieving accurate prediction of glioma molecular typing (IDH genotype, 1p/19q co-deletion, and TERT promoter mutation status), progressive-free survival and overall survival regardless of glioma grades.

In addition to the grade, heterogeneity, and molecular typing of gliomas that are closely related to prognosis, relevant clinical factors such as patient age and performance status may also affect prognosis. Feng et al. [98] used a 3D U-Net-based deep learning model to segment and extract features from preoperative multimodal MRI images of patients with gliomas and combined the extracted radiomics features and clinical features to establish a linear model to predict overall survival in patients with gliomas, and it was indicated that the model had high prediction accuracy in both LGGs and GBMs. Zhang et al. [99] constructed a radiomics nomogram for assessing survival in patients with GBMs using a combination of radiomics features extracted from multi-parametric MRI and clinical risk factors, and the result showed that the consistency indices in the training and validation sets were 0.971 and 0.974, respectively. Huang et al. [100] used CNN to extract deep features from preoperative MRI of patients with gliomas and performed dimensionality reduction, and then they combined dimensionality reduction features with clinical features such as age and tumor grade to build an RF model for survival prediction, and the result was satisfying. 

Most of the studies on the survival prediction of patients with gliomas are retrospective, and the postoperative treatment plans of the enrolled patients are not uniform. In addition, many factors affect the survival of patients. Except for the factors mentioned in the study, some factors are difficult to evaluate, such as psychological factors and postoperative nutritional status. Therefore, patients with a unified treatment regimen should be prospectively included in future studies, and it can also be combined with genomics, metabolomics, and other information to enhance the ability to distinguish the internal information of tumors and improve the ability of survival prediction.

## 6. Limitations and Prospects

With the development and progress of pathological diagnosis technology, people have a clearer understanding of the genetic background and the mechanism of occurrence and development of gliomas. More and more molecular biomarkers have been confirmed to play important roles in the classification, grading, treatment, and prognosis of gliomas. Therefore, the latest WHO classification of tumors of the CNS introduced a series of molecular diagnostic indicators on the basis of histological diagnosis, put forward the concept of integrated diagnosis, and promoted the diagnosis and treatment of gliomas in the era of precision medicine. Radiomics has played an important role in this process, and related research has also made some progress, but the following problems still exist. First, since most of the image data used in radiomics studies come from different centers, corresponding to different equipment and different operators, there is a lack of uniform standards, resulting in differences in the collected images. Secondly, delineating the ROI on the collected images is one of the most critical steps in radiomics research, and there may be deviations between different manually marked lesion areas, which in turn affects the accuracy of the prediction model. Thirdly, most radiomics studies are retrospective, and limited conclusions can be drawn. In addition, machine learning has a huge demand for data sample size, and the lack of sample size will directly affect the accuracy and credibility of prediction results.

Therefore, in future radiomics research, it is necessary to adopt a standardized image acquisition method to obtain imaging data, so as to ensure the reliability and consistency of the data. Meanwhile, deep learning technology should be applied to image segmentation, and the ROI ought to be delineated and segmented under the setting of unified parameters to ensure the accuracy of the prediction models. Furthermore, it is necessary to carry out more large-scale prospective research with multi-center participation to apply and improve the prediction models in clinical practice continuously. Finally, trying to combine pathology, genomics, and radiomics to improve the accuracy of prediction models.

## 7. Conclusions

Radiomics improves the utilization of medical imaging data, enables accurate identification of adult gliomas from other intracranial tumors in a non-invasive manner, and has demonstrated excellent performance in predicting the grade, genotyping, and prognosis of adult gliomas, providing a scientific basis for the clinical application of the individualized diagnosis and treatment model. There are grounds to believe that the combination of radiomics and more advanced technologies will make greater contributions to the development of precise diagnosis and treatment of gliomas in the future.

## Figures and Tables

**Table 1:** Studies Investigating the Role of Radiomics in Differentiation HGGs and SBM.

Authors and Reference No.	Year	Study Sample(*n*)	Imaging Method and Sequence	Feature Extraction/Software	Classification Algorithm	Main Findings
Chen et al. [7]	2019	GBMs and BM (134)	MRI CE-T1WI	Texture analysis/LifeX	Linear discriminant analysis, Logistic regression	AUC 0.80, sensitivity 69%, specificity 86%, accuracy 78%
Artzi et al. [8]	2019	GBMs (212) and BM (227)	MRI CE-T1WI	Multiple features /MatLab (R2017a)	Support vector machine	AUC 0.96, sensitivity 86%, specificity 85%, accuracy 85%
Bae et al. [9]	2020	GBMs (159) and SBM (89)	MRI (T2WI + 3D-CE-T1WI)	Multiple features /Pyradiomics 2.1.0	Adaptive boosting, Support vector machine, Linear discriminant analysis, Deep neural network	The DNN model showed higher diagnostic performance than the traditional machine learning models, with an AUC of 0.956, sensitivity of 91%, specificity of 88%, accuracy of 89%.
Ortiz-Ramón et al. [10]	2020	GBMs (50) and BM (50)	MRI CE-T1WI	Texture analysis /MatLab (R2015b)	Support vector machine	AUC = 0.896 ± 0.067, sensitivity 82%, specificity 80%
Zhang et al. [11]	2021	GBMs (50) and SBM (50)	MRI (CE-T1WI +T2WI + DWI/ADC) + ^18^F-FDG PET/CT	Multiple features /Not mentioned	Random forest	The integrated radiomics model showed more efficient diagnostic performance than any other single radiomics model (AUC 0.93, sensitivity 83.5%, specificity 84.9%).
Causans et al. [12]	2021	GBMs (71) and BM (72)	MRI 3D-CE-T1WI	Multiple features /PyRadiomics 2.1.2	Logistic regression	AUC 0.85, sensitivity 75%, specificity 86%, accuracy 80%
Su et al. [13]	2021	GBMs (157) and SBM (98)	MRI CE-T1WI	Multiple features/AK software version 3.2.0	Logistic regression	AUC 0.81, sensitivity 85.3%, specificity 72.3%, accuracy 76.3%
Sartoretti et al. [14]	2021	GBMs (21) and BM (27)	MRI APTWI	Multiple features/3D Slicer (v. 4.10.2) with PyRadiomics package	Multiple perceptron	AUC 0.836, sensitivity 81.3%, specificity 81.1%
Marginean et al. [15]	2022	HGGs (17) and SBM (19)	CT CECT	Texture analysis /MaZda version 5	Multiple regression	Seven texture parameters were able to differentiate between HGGs and BMs with variable sensitivity (56.67–96.67%) and specificity (69.23–100%).
Cao et al. [16]	2022	GBMs (50) and SBM (50)	MRI (CE-T1WI + T2WI) + ^18^F-FDG PET/CT	Multiple features /Python’s PyRadiomics package	Support vector machine, Logistic regression, K nearest neighbors, Random forest, Adaptive boosting	The model set based on MRI combined with ^18^F-FDG-PET had the highest average AUC (0.93) compared with isolated MRI or 18F-FDG-PET.

**Table 2:** Studies Investigating the Role of Radiomics in Preoperative Grading Adult Gliomas.

Authors and Reference No.	Year	Study Sample(*n*)	Imaging Method/Sequence	Feature Extraction/Software	Classification Algorithm	Main Findings
Chen et al. [21]	2018	HGGs (220) and LGGs (54)	T1WI + CE-T1WI + T2WI + T2-FLAIR	Multiple features /Pyradiomics	Support vector machine	Accuracy 91.27%, weighted macroprecision 91.27%, weighted macrorecall 91.27%
Tian et al. [22]	2018	HGGs (111) and LGGs (42)	T1WI + CE-T1WI + T2WI + DWI/ADC + 3D-ASL	Texture analysis /MatLab (R2012b)	Support vector machine	AUC 0.987, accuracy 96.8% for classifying LGGs from HGGs; AUC 0.992, accuracy 98.1% for classifying grades III from IV.
Jeong et al. [23]	2019	HGGs (13) and LGGs (12)	DSC-MRI	Multiple features /Imaging Biomarker Explorer	Random forest	AUC was 0.94 and the mean prediction accuracy was 0.950 ± 0.091 for HGG and 0.850 ± 0.255 for LGG.
Park et al. [24]	2019	LGGs 204	CE-T1WI + T2WI + T2-FLAIR	Multiple features /Pyradiomics 1.2.0	Elastic net, Random forest, Gradient boosting machine, Linear discriminant analysis	The performance of the best classifier was good in the internal validation set (AUC, 0.85) and fair in the external validation set (AUC, 0.72) to predict the LGG grade.
Nakamoto et al. [25]	2019	HGGs 224 (WHO III 77, IV 147)	CE-T1WI + T2WI	Multiple features /Open-source MATLAB code	Logistic regression, Support vector machine, Standard neural network, Random forest, Naïve Bayes	The mean AUC value for all prediction models constructed by the machine learning algorithms in the LOOCV of the primary dataset was 0.902 ± 0.024. In the independent validation, the mean AUC value for all prediction models was 0.747 ± 0.034.
Haubold et al. [26]	2020	Gliomas 30 (WHO 1 1, 2 13, 3 7, 4 9)	^18^F-FET PET-MRI	Multiple features /Pyradiomics	Support vector machine, Random forest	The AUC of differentiating low-grade glioma vs. high-grade glioma was 85.2%.
Zhang et al. [27]	2020	HGGs (65) and LGGs (43)	DTI	Multiple features /Matlab 2016b	Support vector machine	AUC 0.93, accuracy 0.94, sensitivity 0.98, and specificity 0.86 in classifying LGG from HGG, while AUC 0.99, accuracy 0.98, sensitivity 0.98, and specificity 1.00 in classifying grade III from IV.
Gutta et al. [28]	2021	Gliomas 237 (WHO I 17, II 59, III 46, IV 115)	T1WI + CE-T1WI + T2WI + T2-FLAIR	Multiple features /Pyradiomics	Convolutional neural networks, Support vector machine, Random forests, Gradient boosting	Using learned features extracted from the convolutional neural network achieved an average accuracy of 87%, outperforming the methods considering radiomic features alone.
Su et al. [29]	2021	Gliomas 139 (WHO I 2, II 67, III 36, IV 34)	FLAIR + DWI/ADC + DKI	Multiple features /MATLAB platform (v13.0)	Adjusted-imbalanced Logistic regression	The combination of all multi-parameter MRI radiomics features performed the best predictive AUC (0.853) for differentiating low-/high-grade gliomas.
Cheng et al. [30]	2021	HGGs (210) and LGGs (75)	T1WI + CE-T1WI + T2WI + T2-FLAIR	Multiple features /PyRadiomics toolbox	Logistic regression, Support vector machine, Random forest, XGBoost	The radiomic signatures utilizing the features of intratumoral volume and peritumoral volume both showed a high accuracy in predicting glioma grade, with AUCs reaching 0.968.
Ning et al. [31]	2021	HGGs (211) and LGGs (356)	CE-T1WI + T2-FLAIR	Multiple features /Python 3.6	Support vector machine	The AUC, sensitivity, and specificity of the model based on a combination of radiomics and deep features were 0.94, 86%, and 92%, respectively, for the validation cohort.
Ding et al. [32]	2022	HGGs (68) and LGGs (83)	CE-T1WI	Multiple features /PyRadiomics 3.0.1	Support vector machine, Random forest, Logistic regression	The optimal model was a random forest model that combined radiomic features and VGG16 deep learning features derived from multiplanar CE-T1W MPR images, which achieved an AUC of 0.847 in the training cohort and 0.898 in the test cohort.
Lin et al. [33]	2022	HGGs (50) and LGGs (50)	T1WI + CE-T1WI + T2WI + DWI/ADC + ^1^H-MRS + DTI	Multiple features /Analysis-Kit	Logistic regression	CE-T1WI exhibited the highest grading efficacy among single sequences (AUC 0.92; sensitivity 0.89; specificity 0.85), but the efficacy of the combined model was higher (AUC 0.97; sensitivity 0.94; specificity 0.91).

**Table 3:** Studies Investigating the Role of Radiomics in Predicting IDH Mutation Status.

Authors and Reference No.	Year	Study Sample (*n*)	Clinical Information Included	Imaging Method/Sequence	Feature Extraction/Software	Classification Algorithm	Main Findings
Lohmann et al. [39]	2018	Gliomas 84 (IDH mut 26, IDH wt 58)	No	FET-PET MRI	Texture analysis /LIFEx 2.2	Logistic regression	The overall accuracy of the model (combination of standard PET parameters with textural features) was 82% after 5-fold cross-validation and 86% after 10-fold cross-validation.
Li et al. [40]	2018	IDH1 mut (20), IDH1 wt (205)	Yes	T1WI + CE-T1WI + T2WI + T2-FLAIR	Multiple features/In-house Matlab program	Random forest	The model combining all-region imaging features with age achieved the best performance of accuracy of 97%, AUC 0.96.
Li et al. [41]	2019	IDH mut (51), IDH wt (76)	Yes	^18^F-FDG PET/CT	Multiple features /PyRadiomics	Logistic regression	The generated radiomic signature with the incorporation of age and type of tumor metabolism achieved AUCs of 0.911 and 0.900 in the training and validation cohorts, respectively.
Liu et al. [42]	2019	LGGs 158 (IDH mut 118, IDH wt 40)	No	T2WI	Multiple features /MATLAB 2014a	Logistic regression	Using a classification model of 86 radiomic features, the enrolled patients were correctly classified into the IDH wt and the IDH mut groups (AUC = 1.00).
Tan et al. [43]	2019	Astrocytomas 105 (IDH mut 51, IDH wt 54)	Yes	CE-T1WI + T2-FLAIR + DWI/ADC	Multiple features /Not mentioned	Support vector machine	The radiomics nomogram based on the radiomics signature and age performed better than the clinico-radiological model (training cohort, AUC = 0.913 and 0.817; validation cohort, AUC = 0.900 and 0.804).
Wu et al. [44]	2019	Gliomas 126 (IDH mut 39, IDH wt 87)	No	T1WI + CE-T1WI + T2WI + T2-FLAIR	Multiple features /R software (version 3.3.1)	Support vector machine, Random forest, Adaptive boosting, Naive Bayes, Flexible discriminant analysis, k-Nearest neighbors, Neural network	Random forest showed the highest predictive performance (accuracy 0.885 ± 0.041, AUC 0.931 ± 0.036).
Park et al. [45]	2020	LGGs 168 (IDH mut 113, IDH wt 55)	No	DTI + CE-T1WI + T2WI + T2-FLAIR	Multiple features /PyRadiomics	Random forest	Adding DTI radiomics to conventional radiomics significantly improved the accuracy of IDH status subtyping (AUC 0.900, *p* = 0.006).
Peng et al. [46]	2020	IDH mut (50), IDH wt (55)	No	CE-T1WI + T2WI + ASL	Multiple features /Pyradiomics	Support vector machine	The accuracy and AUC of the classifier, which combines the features of all three sequences, achieved 82.3% and 0.770 (*p* < 0.05), respectively.
Niu et al. [47]	2020	HGGs 182 (IDH mut 79, IDH wt 103)	No	CE-T1WI	Multiple features /Analysis Kit	Logistic regression	The radiomic model showed good discrimination in both the primary dataset (AUC 0.87, sensitivity 85.5%, specificity 75.4%) and the validation dataset (AUC 0.86, sensitivity 91.3%, specificity 69.0%).
Tan et al. [48]	2020	Astrocytomas 62 (IDH mut 30, IDH wt 32)	Yes	DKI + DTI	Multiple features /Not mentioned	Logistic regression	The radiomics model built using the three most informative radiomics features for each genotype yielded an AUC of 0.831 for predicting IDH genotype.
Manikis et al. [49]	2021	IDH mut (41), IDH wt (119)	No	DSC-MRI	Multiple features /Pyradiomics	Support vector machine, Random forest, K-nearest neighbor, Logistic regression, L1 norm penalties, Decision trees	The maximum performance of the IDH mutation status prediction on the validation set had an accuracy of 70.6% (AUC 0.667, sensitivity 60%, specificity 73.6%) when dynamic-based standardization of the images was performed prior to the radiomics.
Choi et al. [50]	2021	Gliomas 1166 (grades II–IV)	No	CE-T1WI + T2WI + T2-FLAIR	Multiple features /PyRadiomics 2.2.0	Convolutional neural network	The hybrid model achieved accuracies of 93.8%, 87.9%, and 78.8%, with AUCs of 0.96, 0.94, and 0.86 in the internal test, SNUH, and TCIA sets, respectively.
Zaragori et al. [51]	2022	Gliomas 72 (IDH mut 43, IDH wt 29)	No	^18^F-FDOPA PET/CT	Multiple features /Pyradiomics	Logistic regression, Neural networks, Random forest, Support vector machine	The combination of logistic regression with L2 regularization and 5 selected features was the best-performing model for predicting IDH mutations and yielded an AUC of 0.831.

**Table 4:** Studies Investigating the Role of Radiomics in Predicting MGMT Promoter Methylation Status.

Authors and Reference No.	Year	Study Sample (*n*)	Clinical Information Included	Imaging Method/Sequence	Feature Extraction/Software	Classification Algorithm	Main Findings
Xi et al. [53]	2018	GBMs 98 (MGMT methylated 48, unmethylated 50)	No	T1WI + CE-T1WI + T2WI	Multiple features /MatLab 2014a	Support vector machine	The best classification system for predicting MGMT promoter methylation status originated from the combination of 36 T1WI, T2WI, and CE-T1WI image features, with an accuracy of 86.59%.
Li et al. [54]	2018	GBMs 193 (MGMT methylated 86, unmethylated 107)	Yes	T1WI + CE-T1WI + T2WI + T2-FLAIR	Multiple features /R package Boruta	Random forest	The radiomics model with six all-relevant features allowed pretreatment prediction of MGMT methylation (AUC = 0.88, accuracy = 80%).
Jiang et al. [55]	2019	LGGs 122 (MGMT methylated 86, unmethylated 107)	No	3D CE-T1WI + T2WI	Multiple features /PyRadiomics 2.1.0	Support vector machine, Random forest, AdaBoost	The fusion radiomics model, which was constructed from the concatenation of both series, displayed the best performance, with an accuracy of 84.9% and an AUC of 0.970 in the training dataset, and an accuracy of 88.6% and an AUC of 0.898 in the validation dataset.
Wei et al. [56]	2019	Astrocytomas 105 (MGMT methylated 73, unmethylated 32)	Yes	CE-T1WI + T2-FLAIR + ADC	Multiple features /PyRadiomics	Logistic regression	The fusion radiomics signature exhibited supreme power for predicting MGMT promoter methylation, with AUCs of 0.925 in the training cohort and 0.902 in the validation cohort.
Kong et al. [57]	2019	Gliomas 107 (MGMT methylated 59, unmethylated 48)	Yes	^18^F-FDG-PET/CT	Multiple features /PyRadiomics	Support vector machine, Logistic regression	The radiomics signature displayed the best performance with AUCs reaching 0.94 and 0.86 in the primary and validation cohorts, respectively, which outweigh the performances of the clinical signature and fusion signature.
Crisi et al. [58]	2020	GBMs 59 (MGMT methylated 20, unmethylated 39)	No	DSC-MRI	Multiple features /LIFEx	Naive Bayes, Decision trees, Multilayer perceptron	The model formulated by multilayer perceptron machine learning methods performed well with 75% sensitivity, 85% specificity, and an AUC of 0.84.
Qian et al. [59]	2020	GBMs 69 (MGMT methylated 26, unmethylated 43)	No	^18^F-DOPA-PET/CT	Multiple features /PyRadiomics	Extra trees, Support vector machine, Random forest, XGBoost, Neural network	The Random Forest model based on features extracted HGG contour alone achieved 80% ± 10% accuracy for 95% confidence level in predicting MGMT status.
Huang et al. [60]	2021	Gliomas 53 (MGMT methylated 21, unmethylated 32)	Yes	T1WI + CE-T1WI + T2WI + T2-FLAIR	Texture analysis /Analysis Kit	Logistic regression	The AUCs for the combined model based on Radscores were 0.818, with 90.5% sensitivity and 72.7% specificity, in the GBM dataset, and 0.833, with 70.2% sensitivity and 90.6% specificity, in the overall gliomas dataset.

## Data Availability

The data presented in this study are available on request from the corresponding author.
